# Exploring youth sport coaches’ perceptions of intended outcomes of leadership behaviours

**DOI:** 10.1177/17479541221076247

**Published:** 2022-02-17

**Authors:** Matthew EC McGuckin, Jennifer Turnnidge, Mark W Bruner, Jordan S Lefebvre, Jean Côté

**Affiliations:** Reviewers: Gordon Bloom (McGill University, Canada) Fernando Ferreira dos Santos (Polytechnic Institute of Porto, Portugal); 1 School of Kinesiology and Health Studies, 152964 Queen’s University, Kingston, Canada; 2 Department of Physical and Health Education, 6057 Nipissing University, North Bay, Canada; 3 School of Human Movement and Nutrition Sciences, University of Queensland, Brisbane, Australia

**Keywords:** Coach-athlete relationship, full-range leadership model, positive youth development, transformational leadership

## Abstract

Youth sport coaches play an important role in shaping the sport experience and positive development of youth athletes. One salient approach to examining how the interpersonal behaviours of coaches influence young athletes has been through the lens of leadership theories. However, there is limited understanding surrounding the underlying processes associated with coach leadership behaviours, such as how and why behaviours are applied. This study sought to examine *why* do youth sport coaches use specific leadership behaviours and *what* are their intended outcomes when using these behaviours? Eleven coaches (*M_age_* = 42.3, *SD* = 14.5) were recruited from competitive youth soccer and volleyball clubs (athletes’ *M_age_* = 15.8, *SD* *=* 1.9). Coaches participated in a video-stimulated recall interview, during which coaches reflected upon their own coaching behaviours and provided insight into the application of leadership behaviours in youth sport. Findings of the study revealed that contrasting leadership behaviours (e.g. transformational vs. transactional) are associated with distinctive coach objectives (promoting confidence vs. establishing respect). This study can serve to facilitate knowledge mobilization by strengthening our understanding of coaches’ intentions for the implementation of leadership behaviours in a real-world setting.

There is a rich history of literature, stemming from the work of Smith and Smoll,^1,2^ that supports the contention that youth sport coaches play an important role in shaping the sport experience and development of youth athletes.^3^ This has prompted the generation of numerous frameworks guiding the coaches role in athlete development.^4–6^ As expressed by Bloom et al.,^3^ “coaches are increasingly called upon to create environments that foster positive developmental outcomes in youth athletes” (p. 144). Positive youth development is a strength-based approach to youth psychosocial development grounded in the positive psychology movement.^7,8^ In sport, positive youth development has been conceptualized to include the following four desirable developmental outcomes: competence, confidence, connection, and character (i.e. the 4 Cs).^5^ According to Côté and Gilbert's^9^ definition of coaching effectiveness, when acting in appropriate coaching contexts, effective coaches contribute to these positive developmental outcomes.^9^ Moreover, Côté and Gilbert contend that performance demands (i.e. competitive level) and developmental level (i.e. age and maturity) are the most important variables in defining a specific coaching context.^9^

Decades of research across the globe supports the role of coaches in fostering the positive development of youth athletes.^1–12^ For instance, youth sport coaches who form individualized, supportive relationships with athletes can enhance the development of social skills and personal assets (e.g. character, confidence).^13^ Coaches can also positively influence developmental outcomes and overall quality of youth sport experiences by structuring physical and social environments that foster quality relationships (i.e. connection).^13^ Similarly, coach-athlete relationship quality has been associated with several significant outcomes for both coaches and athletes, ranging from coaches’ subjective wellbeing,^14^ to athletes’ intrinsic motivation,^15^ improved performance,^16^ and satisfaction with training and performance (i.e. competence).^17^ The movement towards utilizing interpersonal coaching behaviours has been gaining considerable momentum in sport psychology research.^18,19^ Taken together, there is ample evidence to suggest that coaches can meaningfully contribute to the process of youth development. One salient approach to examining how the interpersonal behaviours of coaches influence young athletes is through the lens of leadership theory.

According to Vella et al.,^20^ coach leadership is defined as “a process of interpersonal influence that is dependent upon the relationship between coach and athlete, and is used to facilitate the athlete outcomes of competence, confidence, connection, and chatacter” (p. 432). Given the significant role of coaches, researchers have attempted to analyze and conceptualize the coaching process in order to identify effective coaching behaviours. Notably, seminal lines of coaching research have proposed concepts of coaching leadership including the multidimensional model of leadership,^21,22^ mastery-oriented climates,^23^ and autonomy-supportive coaching.^24^ Although these previous concepts have been successfully employed, each has limitations. For example, concerns have been raised regarding the multidimensional model of leadership's applicability within the youth sport context, the predominant use of self-report questionnaires, and the comprehensiveness of the leadership behaviours within the model. Second, the foundational work from Smith, Smoll, and others regarding mastery-oriented climates focuses mainly on professional coaching behaviours (e.g. technical instruction, positive feedback, and promoting mastery of skills) and does not fully capture a wide range of interpersonal coaching behaviours.^18^ Autonomy-supportive coaching has shed light on the influence of motivational outcomes and basic psychological needs, but studies examining the links between autonomy-supportive behaviours and Positive Youth Development (PYD) outcomes remain limited. It is also important to acknowledge that the autonomy-supportive framework is motivational, rather than leadership-based.

Alternatively, one concept that has been gaining significant momentum in the youth sport domain is Transformational Leadership (TFL).^25^ TFL integrates many elements of existing models and frameworks of effective coaching leadership (e.g. importance of interpersonal relationships, promoting autonomy, providing choice, and increasing motivation). Additionally, TFL includes other constructs that have been notably absent from previous coaching leadership frameworks (e.g. moral components). TFL was originally described by James Macgregor Burns (1978)^26^ and has since been tested across several domains (e.g. organizational, military, healthcare, and sport settings). Originally developed in organizational psychology, TFL falls within the full-range leadership model,^27^ which comprises three leadership styles: laissez-faire, transactional, and transformational. An additional leadership style that has recently been identified in sport research is toxic leadership.^28^ Moving along the full-range leadership model in order of most effective (transformational) to least effective (toxic), each leadership style is decribed below.

*Transformational leaders* facilitate personal development among followers via the 4 I's described by Bass and Riggio^29^ (e.g. idealized influence, inspirational motivation, intellectual stimulation, and individualized consideration). Specifically, idealized influence is characterized by behaviours where the leader is conveyed as a positive role model (e.g. discussing or modelling pro-social values such as empathy, morality, and responsibility). Inspirational motivation is characterized by behaviours through which the leader demonstrates that they hold high expectations or a compelling vision for the future of their followers (e.g. expressing confidence in follower potential). Intellectual stimulation is portrayed through behaviours that encourage followers to think or convey a view of followers as capable decision makers and collaborators (e.g. eliciting input from followers, sharing decision making responsibilities or emphasizing the learning process). Lastly, individualized consideration is displayed via behaviours through which a coach recognizes the followers individual needs and displays genuine care or concern (e.g. discussing personal issues, adapting tasks to suit the followers needs, or recognizing individual contributions). The fundamental tenet of TFL theory is that by using these styles of interaction, transformational leaders foster higher levels of follower performance and personal growth.^27^ While it is important to note that transactional leadership can be described as the foundation of coaching, it is the leader who displays TFL in conjunction with transactional leadership that will optimize growth and development among followers via the augmentation effect.^27^ The augmentation effect stipulates that measures of transformational leadership add to measures of transactional leadership in predicting outcomes, but not vice versa.^27^

*Transactional leadership* falls within the middle of the leadership continuum, whereby leaders facilitate motivation and performance through rewarding followers for meeting goals and objectives as well as correcting them for failing to meet objectives.^30^ For example, providing punishments for undesireable follower behaviours or rewards for expected behaviours are considered transactional leadership behaviours. Next, *laissez-faire leadership* is characterized by leader absence or lack of involvement and is generally viewed as passive and ineffective.^29^ Examples of laissez-faire behaviours include showing disinterest or avoiding action towards followers. Lastly, *toxic leadership* was originally conceptualized in militaristic settings and has since been recognized as a component of coaching leadership.^28,31^ Essentially, toxic leadership consists of behaviours wherein coaches display negative attitudes or frustration towards athletes. As examples, using a negative tone, sarcasm, and displaying anti-social behaviours are considered forms of toxic leadership behaviours.

TFL theory has shown promise as a viable avenue for enhancing sport experiences.^31^ In their critical review of TFL in sport, Arthur and colleagues came to the conclusion that TFL theory offers a relevant framework for the coaching context in sport. Moreover, early studies examining the effects of TFL on athlete outcomes offer positive discoveries such as increases in athletes’ motivation, team-level performance and cohesion,^32,33^ facilitation of developmental outcomes,^33^ basic needs satisfaction,^34^ and expedited developmental experiences.^35^ However, the majority of initial studies aimed at testing the applicability of TFL in youth sport utilized self-report, questionnaire-based data collection methods, with few exceptions.^36,37^ Even still, the few qualtitative studies that exist have measured only athlete perceptions regarding transformational leadership behaviours and focused on elite sport settings. Specifically, both Newland and colleagues^36^ and Smith et al.^37^ focused on describing what transformational behaviours look like in sport settings by interviewing elite cricket players^37^ and collegiate basketball players.^36^ The findings of both studies served as an important initial step towards understanding what TFL behaviours might look like in sport by gathering follower perceptions of transformational behaviours that were used by team leaders (i.e. coaches and captains).

As a result of the overreliance of correlational studies using self-report questionnaires, researchers have advocated for the examination of coach leadership using methodologies, such as systematic observation.^38–40^ For instance, the Coach Leadership Assessment System (CLAS)^40^ is an observation coding system which allows for coding of specific, observable coaching behaviours categorized across the full-range leadership model. Lefebvre et al.^39^ used the CLAS to quantify behaviours across the full-range leadership model among youth sport coaches and discovered that coaches more frequently displayed behaviours coded as individualized consideration and inspirational motivation compared to intellectual stimulation and idealized influence. The findings of their study provided broad perspective regarding the objective measurement of leadership behaviours employed by youth sport coaches.^39^

Additionally, investigators have gone so far as to develop and test interventions for transformational coaching in youth sport.^38,40^ One group of researchers^41^ outlined and evaluated a novel transformational coaching workshop.^38^ Coaches participated in a three-hour transformational coaching workshop, which focused on educating coaches about specific behaviours within the full-range leadership model via lecture, videos, and group discussion. Systematic observation of each participant (n = 8) using the CLAS^40^ occurred pre- and post-intervention. The results of the observation identified that coaches more frequently displayed three of the four transformational behaviours, and spent less time simply observing athletes following the intervention. Notably, this was the first intervention to use data collection procedures that did not involve questionnaires measuring athlete or coach perceptions.

Certainly, considerable advances have been made towards applying the full-range leadership model and TFL behaviours in youth sport, however notable gaps in this literature still remain. First, there has been a relative absence from the voices of youth sport coaches, those who are intended to use TFL as a means towards effective coaching. This is one of a few studies to gather coach perceptions pertaining TFL behaviours.^41,42^ Secondly, Arthur and colleagues^31^ suppose that there have been fundamental shortcomings in the definition of TFL theory as outcome rather than behaviour: “the assertion that transformational leaders behave in ways ‘to achieve superior results’, ‘that motivate and inspire those around them’ or that ‘stimulate followers’ efforts to be innovative and creative’ describes transformational leaders by their outstanding outcomes on followers, and makes a test of the construct true by definition” (pp.7–8).^31^ In general, studies examining the general underlying processes associated with coach behaviours (e.g. how and why behaviours are applied) are limited^32,33^ and to our knowledge, none have examined this process in relation to leadership. Accordingly, the purpose of this study was to examine the underlying processes of coach leadership behaviours. Specifically, this study sought to examine *why* do youth sport coaches use specific leadership behaviours and *what* are their intended outcomes when using these behaviours? Given the aforementioned methodological limitations within the coach leadership literautre, the purpose was accomplished by examining the perceptions of youth sport coaches using a video-stimulated recall procedure.

## Methods

### Methodological orientation

The study was situated within a post-positivist philosophical paradigm, which was underpinned by a critical realist ontology and a modified dualist/objectivist epistemology.^43–45^ Critical realism assumes the existence of one universal reality independent of the individual that can be closely approximated but never fully understood.^45^ A modified dualist/objectivist epistemology acknowledges that the research process plays a role in the construction of knowledge, however this influence should be minimized in attempt to reduce bias.^45^ Accordingly, the methods of our study attempt to mitigate bias and maintain objectivity through a number of techniques. As an example, the methods used in this study attempted to triangulate participant experiences (i.e. interviews) in conjuction with objective data (i.e. video-observation). Furthermore, techniques used throughout the data analysis (i.e. inter-rater reliability) were used enhanced the objectivity of the findings.

Video-stimulated recall is a mixed-methods protocol defined as “an introspection procedure in which videotaped passages of behaviours are replayed to individuals to stimulate recollection of their concurrent cognitive activity” (p. 861).^46^ Similar interviewing protocols have been previously used to explore youth sport coaches’ experiences.^47,48^ In fact, researchers have noted that this type of research method has potential for examining contexts that are uncertain and characterized by non-deliberative behaviour, similar to that of the coaching context.^46^ Furthermore, Turnnidge and Côté^40^ highlighted the need for qualitative and observational methods to complement quantitative methods that continue to be employed. Stimulated recall interviewing is a technique that fullfills this need by tapping into coaches’ perceptions of why they engaged in a particular behaviour.

### Participants

Prior to the beginning of the study, institutional review board granted approval and consent was obtained from all participants. Coaches were recruited through convenience sampling, and were required to meet eligibility criteria (listed below) in order to take part in the study. Participating coaches were required to have a minimum coaching certification for their governing sport body, two or more years of youth sport coaching experience. Furthermore, all participants were coaching athletes ranging from 13–18 years of age in soccer (n = 2 female teams, 3 male teams) and volleyball (n = 2 female teams, 3 male teams). The competitive teams were regional travelling teams who had more than one practice and game per week. The purpose for the eligibility criteria was to increase the likelihood that coaches had a range of experiences to draw upon during their interviews. Participants were recruited from competitive soccer (n = 2 female teams, 3 male teams) and volleyball (n = 2 female teams, 3 male teams) youth sport programmes. A total of 20 coaches were initially contacted, of which 11 agreed to participate for the length of the study. All participants were male ranging from 19 to 68 years of age *(M_age_* = 42.3*, SD* = 14.5*)* with an average of 11.5 years of coaching experience (*SD* = 15)*.* Consent was obtained from the coaches, athletes, and parents prior to data collection.

### Procedure

**Figure 1.**
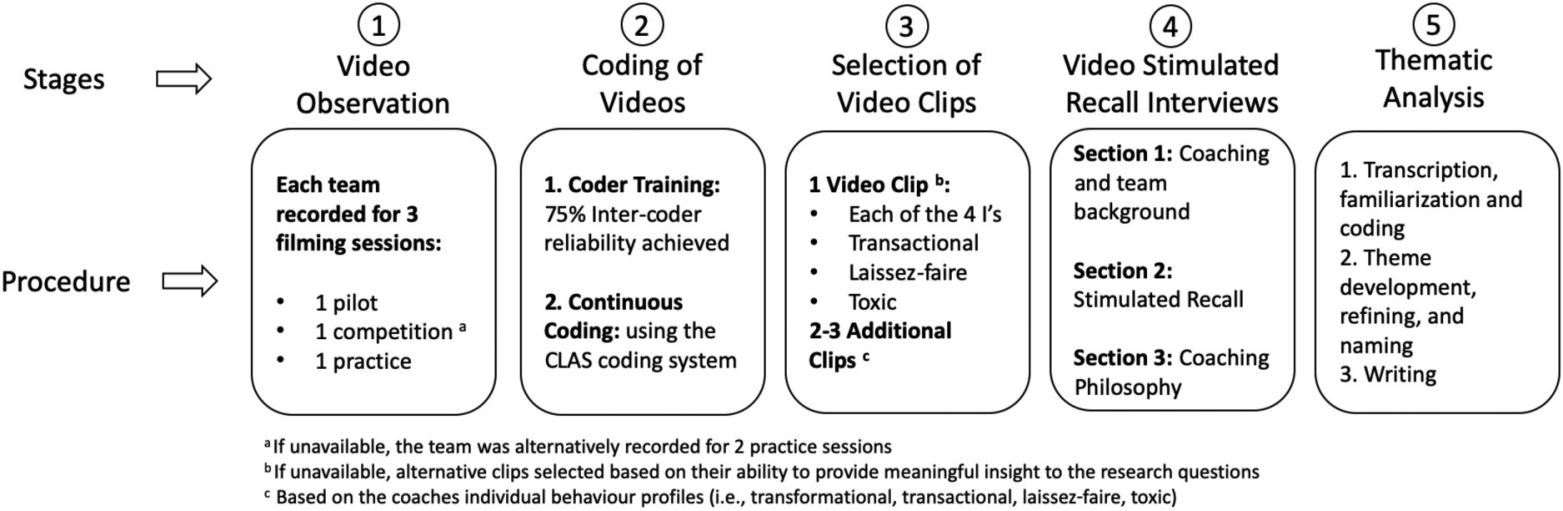
Outline of study design.

The details of the data collection procedure are outlined in Figure 1. Initially, observational data was collected for each team at three time points during the season; once at the beginning, once in the middle, and once at the end (Figure 1, Stage 1). The first training session served as a pilot session to allow the participants to become acclimated to the cameras. Two cameras were used for each observation; one camera captured a wide angle of the entire play area, while the other was manually operated to capture coach behaviours and interactions with athletes. Coaches wore a lapel microphone and a large parabolic microphone was operated by researchers to capture audio from both coaches and athletes. The three filming sessions included two training sessions and one competition when possible. Coaches who did not have a competition in the proximal area of the research (n = 3), were alternatively filmed for one pilot session and two practice sessions. Videotaping sessions lasted between 90 to 120 min resulting in more than 40 h of video data. Capturing data from multiple contexts and time points enabled the researchers to gain a richer sample of observational data.

Following data collection, coach leadership behaviours were coded using the CLAS (Figure 1, Stage 2).^40^ The system classifies coach behaviours according to the interactive content and leadership tone—broadly classifying TFL (11 codes, which can be further divided into the four I's), for example: (a) *expressing anger and hostility* (Toxic), (b) *showing disinterest* (Laissez-faire), (c) *discussing rewards and penalties* (Transactional), (d) *showing vulnerability or humility* (Idealized Influence), (e) *expressing confidence in athlete potential* (Inspirational Motivation), (f) *sharing decision making and leadership responsibilities* (Intellectual Stimulation), and (g) *showing interest in individual athlete feelings, needs, or concerns* (Individualized Consideration). In total, the CLAS is comprised of 17 specific behaviours, each falling into one of the catergories of the FRLM or alternatively, considered ‘Neutral’ (i.e. giving instructions). Prior to coding the videos for the current study, the primary investigator completed a coder training programme for the CLAS to ensure adequate skill and knowledge of the system.^40^ To complete the training programme, coders: (1) were provided with relevant reading materials, (2) participated in group-based coding sessions, and (3) completed the independent coding of 10 min video segments until inter-rater reliability was obtained. Accordingly, in congruence with Erickson & colleagues (2011) guidelines, the primary investigator achieved a minimum 75% standard for inter-rater reliability on two separate 10-min video segments.^48^ For more detail regarding the coder training and protocols for the CLAS, refer to Turnnidge & Côté.^40^

Following video coding, specific video clips were selected for stimulated recall interviews (Figure 1, Stage 3). Clips were purposefully selected based on their ability to offer meaningful insight into the project's research question.^49^ Each coach was shown 9–10 clips that ranged across as many of the leadership behaviours as possible and were representative of their leadership style (e.g. transformational, transactional, laissez-faire, and toxic). For example, if a coach displayed each of the behaviours across the leadership spectrum, one clip from each leadership style was selected. Furthermore, if a coach frequently displayed certain behaviours (e.g. coded as Inspirational motivation), additional clips of that behaviour were selected. However, if a video clip of a specific behaviour was unavailable for a given coach (e.g. laissez-faire), an alternative video clip was selected that was representative of that coach's behaviour profile (e.g. inspirational motivation). Using these clips, a personalized video compilation was prepared for each coach using iMovie sofware. Clips were compiled chronologically and separated by a five second black screen.

In stage 4 of our procedure, the lead author conducted each interview using a semi-structured interview guide to prompt the participant's responses following the observation of a single video clip (i.e. video-stimulated recall; see Figure 1). Participants were asked to expand upon their responses using probing questions from the interviewer. The primary interview procedure focused on obtaining participants’ perceptions of the following recorded behaviours: (a) their perception of the recorded behaviour (e.g. “Could you explain what is happening in this clip?”), (b) the application of the behaviour in other scenarios (e.g. “At what other times during practices or games might you use this behaviour?”), (c) antecedents to the behaviour (e.g. “If anything, what lead you to this behaviour?”), and (d) intended outcomes of the behaviour (e.g. “What are the specific goals or outcomes that you feel might result from this behaviour?”). Interviews resulted in 733 total minutes of data with a mean length of 66 min (*SD* = 14.4). Interviews were recorded and transcribed verbatim and supplemented by the researcher's notes taken during and following each interview. Pseudonym names were given to each participant in an effort to maintain their anonymity.

### Data analysis

A thematic analysis following the six-phase model outlined by Braun and colleagues^50^ was selected to analyze interview transcripts. The lead author began the process of analysis by reviewing all audio recordings (i.e. familiarization with the data). Next, the lead author and a research assistant transcribed the interviews verbatim and discussed patterns and initial codes (i.e. generating initial codes and searching for themes). During the transcription and review of interviews, the lead author and research assistant agreed on a number of initial codes that were frequently discussed by the participants. Then, the first, second, and fifth authors reviewed these initial codes and collated codes into potential themes (i.e. reviewing themes). Latent line by line coding was completed by the lead author and any confusion regarding specific codes were discussed and resolved with the second author. Codes, sub-themes, and themes were continually reviewed throughout the coding process by comparing them against the entire dataset and discussing them with members of the authorship team. Reviewing the themes ensured that the final themes and sub-themes reflected the essence of the participants experiences in conjunction with the researchers understanding of those experiences. Importantly, the thematic analysis was deductive. That is, the selection of video-clips were derived from the FRLM,^29^ which subsequently informed the semi-structured interviews and the ensuing analysis (i.e. development of themes). In addition, throughout the initial steps of analysis, it became clear that participants distinctly discussed outcomes of transformational behaviours which mapped onto the 4 C's of PYD.^7^ As a result, codes and sub-themes for transformational behaviours were defined and named to reflect these developmental outcomes. As an example, the transcript extract “It's just to get them to be more comfortable around people that they don't know or be more forthcoming and making someone else feel comfortable” was derived from a video clip displaying *intellectual stimulation*. This extract was coded as *prosocial values*, which was combined with other codes to generate the sub-theme “Establish Connection” under the theme “Transformational Leadership”. Taken together, the deductive thematic analysis resulted in three themes, which were comprised of nine subthemes.

### Research quality

Based on reccomendations from Sparkes and Smith,^51^ specific criteria have been selected for this study in an effort to optimize research quality. Following the recommendations of Sparkes and Smith, the list of criteria for this study were tailored to help enhance the quality of the research. Furthermore, the criteria are in line with the research question being examined and include: (a) importance of the research, (b) selection of appropriate methods (c) reflexivity, and (d) credibility. Specifically, providing implications for practice or providing suggestions for future research can deem research as important.^51^ Given its recent attention and application to the sport context, qualitative research is important to inform the application of TFL. In addition, findings from this study helped guide development of a coaching workshop and development programme^42^ through its unique methods. The methods used in this study may be deemed appropriate given their uniqueness as well as the thorough description and documentation of the methods and methodological decisions. Lastly, to enhance researcher reflexivity, a detailed list of all thoughts, feelings, and emotions relating to the research topic, known as an audit trail^52^ was kept and used to remind the primary investigator of any biases or emotion that may have influenced the data or data collection, helping to establish honesty and authenticity of the research. Finally, the use of critical friends (i.e. members of the research team) occurred throughout data analysis process to establish credibility.^51^ Specifically, at each stage of the analysis, members of the research team were consulted in order to help understand participant meanings and construct accurate, meaningful depictions of the data.

## Results

### Intended outcomes of coach leadership behaviours

Coach perceptions of their intended outcomes of behaviours were discussed and summarized according to leadership style (i.e. Transformational, Transactional, and Toxic; Table 1). Although a number of questions were asked pertaining to how and why leadership behaviours were applied by coaches, the findings of the study focus on the discussion surrounding the intended outcomes of these specific behaviours. It should be noted at this point that laissez-faire behaviours were seldomly seen with the coaches in this study and therefore will not be reported in the results but will be addressed in the discussion.

**Table 1.** Intended outcomes of coach leadership behaviour by leadership style.

Behaviour	Intended Outcome	Description
**Transformational Leadership**	*Instilling confidence*	Instilling trust in athlete's skills or decision making
	*Promoting competence*	Teaching concepts Developing an understanding of the game
	*Establishing connection*	Promoting supportive, open relationship
	*Developing character*	Building life skills Overcoming barriers Learning to be successful Promoting sportsmanship
	*Providing enjoyable experiences*	Promoting fun Prolonging engagement
**Transactional Leadership**	*Seeking respect and commitment*	Establishing ‘traditional’ coach-athlete relationships Respecting and adhering to team rules Understanding behavioural consequences Understanding success related consequences
**Toxic**	*Emphasizing winning,* *Promoting Competitiveness,* *Lighting the fire*	Winning is an important component of sport Aggression can be okay in sport Promoting unsportsmanlike play Motivating athletes through toxic behaviours (e.g. yelling)

#### Transformational leadership behaviours

When video clips of transformational behaviours were presented, coaches discussed a wide range of intended outcomes: (a) *instilling confidence, (b) promoting competence, (c) establishing connection, (d) developing character,* and (e) *providing enjoyable experiences*. An important consideration when interpreting these results is that when coaches identified their objectives for each type of transformational behaviour, none of the themes appeared to be mutually exclusive to one or more of the four I's of TFL. For example, a coach discussing a behaviour that was coded as “idealized influence”, may have discussed his intended objective to be any one of these themes discussed within transformational behaviours. Along with the description of the behaviour associated with each participant quote, the coded descriptor of each behaviour according to the CLAS is provided.

**Instilling Confidence.** Within this sub-theme, coaches portrayed that specific transformational behaviours were intended to increase or instil confidence within athletes. As an example, one coach was observed discussing expectations of the athletes during half-time when the team was losing (i.e. discussing goals and expectations; inspirational motivation). When elaborating on this behaviour during the interview the coach said:

…[I am] trying to reinforce success. So if you remember the game, we started off really bad. Usually if the team got a couple goals on us, our energy level went down. (This time) they are still playing but you can just tell from their body language that they are not into it. The game the night before was the first time we have been in a game that we were down and we still had the fire and the energy and we were able to tie it up. Unfortunately, we lost in the final minutes of the game, but up until that point I had not seen that. So I just wanted to reinforce that by saying: “look what you guys did (last game). If you did it against that team you can do it against another team as well.” (Kevin)

As another example, one coach wanted to ensure that a player knew he still had confidence in his ability after the player had missed an easy goal (i.e. expressing confidence in athlete potential; inspirational motivation). In responding to the clip the coach said:

Blake, basically received a pass in front of the net and he was so close to getting a goal. Blake is one of the weaker players on the team, mainly because he just played recreationally before, and we added him to the team last minute, and great kid, very motivated, not necessarily a lot of soccer skills, but getting better every game. He is one of those players who you want to see get a goal because it is just a confidence builder. I didn't want him to feel bad that he missed the goal, but I was really excited that he got so close. So I wanted to make sure that when he came off, he was still motivated to be in that position again. I did not want him to go put his head down and say: “I screwed up that easy goal”. So this is a chance to just really encourage what he did well and not worry that he missed the goal, but in the same breath, just give him a few more pointers. (Keith)

Although both examples in this sub-theme use behaviours coded as inspirational motivation, coaches discussed the concept of instilling confidence when using behaviours related to each of the 4 I's.

**Promoting Competence.** Coaches also discussed how specific transformational coaching behaviours were intended to promote development of sport specific knowledge or skill (i.e. competence). Specifically, one coach was observed asking players to watch the play develop from the sideline and to discuss what they would do in that situation (i.e. eliciting athlete input; intellectual stimulation). When asked about the intended outcome of this behaviour, he stated:

What I am trying to get the boys to understand [in this clip] is that the game is not just about going 100% all the time. They have to learn that there are points where they have to slow it down and be calm. What I was trying to do was give them an understanding. It's not about the results, these guys are learning how to play the game. I have always said that I don't care about the result because there are other factors that can change that, but how you understand the game is more important. (Bruce)

Regardless, whether the behaviour was intended to promote sport-specific or game-related knowledge, coaches identified that a range of transformational behaviours can be employed to promote competence.

**Establishing Connection.** Coaches also acknowledged that certain transformational behaviours can be intended to foster interpersonal relationships with or among athletes (i.e. connection) as well as help athletes foster broader social skills. One coach was observed asking an athlete if everything was going well at school and home (i.e. showing interest in athletes feelings, needs, or concerns; individualized consideration). He discussed that his behaviour was intended to help foster a deeper relationship with the athlete:

I want to open the dialogue as volleyball first, because that is what they are there for, but after that they can open up to different things, whether it is at home or school or whatever is going on. So, I want them to think that they can talk to their coach about something volleyball related and if he was comfortable, he can talk about something else. (Dylan)

Although connection was primarily related to developing the relationship between the coach and the athlete, other coaches noted that behaviours can be targeted at fostering broader social skills and promoting prosocial environments. For example, this coach was asking certain players to provide input regarding the performance of a specific skill (i.e. eliciting athlete input; intellectual stimulation). When he was asked about the intended outcome of this behaviour, the coach reflected: “It's just to get them to be more comfortable around people that they don't know or be more forthcoming and making someone else feel comfortable” (Alexander). The coach acknowledged that certain players are more reluctant to speak up in a group setting and that by encouraging the athlete to talk in front of their peers, can help them develop connection in other settings as well.

**Developing Character.** Another clear message derived from the data is that coaches understand that sport is a vehicle for the development of personal values and character, and that transformational behaviours can be utilized to target these outcomes. Specifically, coaches identified that some transformational behaviours were intended to facilitate the development of positive values and behaviours (i.e. sportsmanship, handling criticism, learning to be successful, and learning to lead). As an example, one soccer coach asked the athletes to acknowledge the efforts of the goalie even after he allowed a late goal (i.e. discussing and modelling prosocial values; idealized influence). When asked about the behaviour, the coach said:

We just lost, I think they just scored a minute before that. But in this case, I do not want the goalie feeling that they let the team down, whether it was a tie or a loss, or in this case its just breeding good sportsmanship within the team. You win as a team and you lose as a team, it's not the goalie's fault whether you won or lost it's just getting in the habit of that. In this case the goalie let the goal in last minute so I did not want him to feel down. Me telling the goalie not to feel down, it delivers one effect, but when the players are going up and saying: “Good job, good effort” it means a lot more. (Keith)

In another example, one coach was observed allowing his athletes to make decisions during practise in a specific drill (i.e. sharing decision making; intellectual stimulation). When discussing this behaviour, the coach said:

We are about to have a one on one competition, so hitter A is against hitter B. At this point, it was actually the setters who were going head to head, and if you are going to end up hitting in this game you need to have a setter who you can rely on and trust If you got a setter who isn't very good, it's really easy in that kind of drill to be saying: “oh it's not my fault it's the setters fault.” So I was giving them the personal responsibility of tailoring their training so that they could choose the best situation for themself, the best thing that was available. Really I just wanted them to have to own the fact that what happened was now entirely on them. (Mike)

Throughout the interviews, coaches discussed many transformational behaviours that they used in an effort to develop character in athletes, as seen in these examples.

**Providing enjoyable experiences.** On the whole, coaches discussed a number of transformational behaviours which were intended to foster immediate enjoyable experiences for athletes. The coaches emphasized the importance of enjoying the game and its impact on having athletes return to sport, and long-term participation. In one example, a coach was observed using humour to promote enjoyment (i.e. modelling pro-social behaviours; idealized influence). The coach discussed the intended outcome of this behaviour by saying:

I was just trying to make the kids enjoy the season, it was going to be a tough season from the very beginning, and in reality my measurement of success was how many came back this year for the tryouts. Probably 85% came back, some kids decided to move on to other things, but they still told me at the end of the season that they enjoyed the season and that their parents enjoyed what I did so, that was a reward in itself. (Kevin)

Perhaps not surprisingly, abundant examples of instances where coaches used transformational behaviours to promote enjoyment were available from this dataset.

#### Transactional leadership behaviours

When discussing their own transactional behaviours, coaches discussed objectives that related to one overarching sub-theme; *seeking respect and commitment*. Similar to that of transformational behaviours, coaches discussed these intended outcomes for a range of behaviours. There was a tremendous amount of overlap between the themes of respect and commitment, therefore they are combined into a single sub-theme.

**Seeking respect and commitment.** Participants discussed a number of concepts related to seeking respect and commitment. Firstly, coaches discussed how transactional behaviours can be used to help establish respect. Coaches discussed how transactional behaviours were intended to establish respect for the coach. For example, one coach was observed having a stern discussion with athletes after they lacked focus at the beginning of practise (i.e. responding to deviations from rules or standards; transactional). When asked about the intended outcome of this behaviour, he said:

I think its really entirely okay for my athletes not to like me. Like I don't need them to be my best friend. I need them to respect me, I need them to buy into what it is that I’m asking them to do. But I can certainly call them on the table for behaviour which wasn't appropriate to the moment. I feel that's no problem at all. (Mike)

When further elaborating on the behaviour, this coach discussed that it is not necessary for his athletes to be his friends. More importantly, they need to understand that he is largely there as a superior, rather than a peer, reinforcing the traditional, hierarchical coach-athlete relationship. In this sense, the intended outcome of this behaviour was distinctly different than the transformational behaviours discussed above.

Another coach discussed the importance of athletes respecting team rules. The coach spoke about his rationale for benching a player who was late for warm-up prior to the game (i.e. discussing rewards and penalties; transactional):

Today I told him: “Hector you were going to start but you’re late and so you’re not going to”… I think they should respect timelines in terms of: “Okay, I’m playing today and I need to be there a half hour before warm up…and that doesn't mean 5 min late, that means I have to be there ready to go.” (Kevin)

When asked about the intended outcome of this specific behaviour, the coach responded, “It worked! Because I don't think he was late after this one.” As can be seen, coaches used transactional behaviours to establish respect and adherence to team rules. In a similar sense, coaches discussed that transactional behaviours might be used to help athletes understand the importance of commitment to the team or game (i.e. level of effort, commitment to the game). In some instances, commitment was viewed as a level of effort, for example, one coach was observed having a long discussion with his players about punishments for lack of effort during practise (i.e. discussing rewards and penalties; transactional). When asked about the behaviour he said:

(The team) behaviour was not where it needed to be and I needed to get their attention. There are times where my response to it would have been just everybody on the end line and we are going to run suicides, and after you run your suicides then I would have said: “man, if you are going to keep making mistakes, this is what we are going to do.” (Bruce)

Elaborating about the intended outcome of this behaviour, the coach responded, “I think they need to understand that you’re going to be punished for things like a lack of trying, but not for trying your best and not having the results…” (Bruce). In most instances, coaches discussed using additional physical activity (e.g. push-ups, sit-ups, running) as consequences for undesirable behaviour and occasionally discussed the need to sit or bench players due to undesirable behaviour (e.g. showing up late to a game). Notably, transactional behaviours were used primarily as a retroactive strategy for managing undesireable behaviour.

#### Toxic leadership behaviours

Video clips for this category were present for only 3 of the 11 coaches, therefore, these initial themes should be considered with caution and certainly require further investigation. Overall, toxic behaviours were intended to promote the outcome of *emphasizing winning, promoting competitiveness, and lighting a fire*.

**Emphasizing winning, promoting competitiveness, and lighting a fire.** When discussing toxic behaviours, coaches identified that they were used to emphasize that winning is important, promote competitiveness towards teammates or other teams, and to light a proverbial fire under their athletes. As one example, a coach was observed telling his athletes to be dishonest with a match official during a game (i.e. modelling anti-social behaviours; toxic). The coach discussed that his behaviour was intended to help athletes understand that winning is important in sport. The coach said:

I think they know that I’m there to win, if I didn't say that and I just said “be honest, I don't care if we win or lose” they would ask if I wanted success. So, that gets across that I still care about winning and that the competitive level needs to be there. (Harry)

Another coach was observed asking a player to be aggressive towards an opponent (i.e. expressing anger or hostility; toxic). When elaborating on the behaviour, the coach explained that this would “tell them that aggressiveness is okay in certain circumstances” (Dylan) and hoped that “he [athlete] knows he's allowed to be aggressive [towards his opponent]” (Dylan). Lastly, coaches used toxic behaviours in order to light a proverbial fire under athletes. Coaches displayed this behaviour primarily by using negative language or tone. When asked about objectives of these behaviours, one coach put it quite simply by saying “The most positive outcome is getting an extra spring in his step when he gets on the field” (Mike). Overall the coaches discussed that these behaviours are important because sport provides opportunities to learn how to be competitive. Although we are aware that coaches often have athletes’ best intentions in mind while coaching, the observation and discussion of these behaviours sheds light on the reality of youth sport experiences, in that, they may not always be focused solely on the developmental needs of athletes.

## Discussion

The purpose of this study was to explore coach perceptions related to why leadership behaviours are applied in the youth sport context. Specifically, this study sought to examine *why* do youth sport coaches use specific leadership behaviours and *what* are their intended outcomes when using these behaviours? The observational data used as part of stimulated recall interviews generated a powerful approach to understanding coach behaviour. The findings of this study provide a more complete depiction of leadership in youth sport by offering an opportunity for coaches to share their perspective.

### Intended outcomes of coach leadership behaviours

The main contribution of the present study was related to the theme of *intended outcomes of coach leadership behaviours*. Coaches associated very specific objectives with a particular coach leadership style (e.g. transformational, transactional, and toxic). Interestingly, we found that coaches targeted outcomes associated with PYD via a wide range of transformational behaviours. Specifically, when reflecting upon their own transformational behaviours, coaches identified five sub-themes related to their intended outcomes: *instilling confidence, promoting competence, establishing connection, developing character,* and *providing enjoyable experience.* The five sub-themes associated with transformational behaviours discussed by coaches in this study map readily onto the 4 C's (i.e. Competence, Confidence, Connection, Character, and Enjoyable experiences) of PYD initially outlined by Lerner et al.^7^ and subsequently adapted for sport.^53^ As a strength-based approach, PYD suggests that all youth are capable of positive, successful, and healthy development.^7^ Several studies have suggested that sport participation can be an effective vehicle for promoting PYD among its participants.^54–56^ To this end, Vierimaa and colleagues^3^ proposed that the 4 Cs within PYD, encompass the numerous positive outcomes associated with sport participation^55,56^ and coaches in the present study confirmed that the 4 Cs are important assets to develop in young athletes.

Moreover, the findings of this study are consistent with findings of empirical studies that identified athlete outcomes specifically associated with transformational behaviours.^32,33,35,57^ Particularly, studies have examined outcomes of coach leadership behaviours as perceived by athletes including: competence,^33^ development of personal and social skills,^55^ self-efficacy,^56^ and intrinsic motivation.^56^ In addition, qualitative research within the sports coaching domain support the findings of this study.^56^ Vella and colleagues interviewed youth sport coaches about which outcomes coaches attempt to influence among athletes and identified the 4 Cs of PYD, in addition to team climate, broader life skills, enjoyment, and psychological capacities. Similarly, coaches in the present study identified that transformational behaviours were intended to promote the constructs of PYD alongside enjoyable experiences. Furthermore, previous researchers have found that coaches stress the importance of facilitating immediate and enjoyable sporting experiences using specific behaviours.^53^ The recent work of Visek and colleagues^58^ in their creation of “fun maps” with youth athletes support the important role that coaches play in creating a *fun* sport experience for athletes. In the present study, the immediate enjoyment of athletes in youth sport was similarly intended to be enhanced by coach behaviours that were related specifically to transformational behaviours.

With regards to transactional behaviours, coaches discussed the sub-theme of intended outcomes: *seeking respect and commitment*. In assessing these intended outcomes compared to those discussed with transformational coach behaviours, it is evident that there are distinct differences. Primarily, when discussing transactional behaviours, coaches discussed topics of team structure, rules, and power dynamics. Coaches revealed that they employed transactional behaviours to help enforce values such as respect for superiors and abiding to team rules. Transactional leadership is deemed as a necessary, but insufficient component of leading.^27^ In fact, it has been suggested that the use of transactional behaviours in conjunction with transformational behaviours result in an augmentation effect, which proposes that “more active forms of transactional leadership (contingent reward and active management-by-exception) represent necessary but insufficient conditions for superior performance and responses among followers”.^57^ This conceptualization of transactional leadership, developed and proposed within organizational psychology, is however strikingly similar to that of the style described by coaches within this study. For example, upon viewing their own transactional behaviours, coaches discussed objectives related to maintaining control of athletes through respect and commitment. Therefore, conceptualizations of transactional leadership in youth sport may mirror those proposed in organizational settings. Although transactional behaviours can be useful for providing extrinsic motivation (e.g. providing rewards) to athletes, coaches in this study viewed transactional leadership as a reactive strategy to managing undesired athlete behaviours which could have negative consequences on athletes’ long term sport engagement.

One notable finding of this study is the lack of representation of laissez-faire leadership behaviours. This is somewhat encouraging and not remarkably surprising. Similarly, both observational^39^ and empirical^33^ studies have found that youth sport coaches are seldomly recorded using laissez-faire behaviours. Although these findings are consistent with previous literature, we recognize that observer bias may have influenced the coaches in this study. Another possible explanation is that the coaches who chose to participate in this study may have been more athlete centred than those who did not. To this end, one could speculate that the coaches who chose not to participate in the study may exhibit more laissez-faire behaviours. Alternatively, it is possible that the population (i.e. competitive youth sport coaches) have been exposed to formal education, or have increased demands (i.e. performance objectives) that lead to the lack of representation of these behaviours. Regardless, it is reassuring that these youth sport coaches are not neglecting their coaching responsibilities, showing disinterest (i.e. laissez-faire behaviours), or routinely engaging in negative behaviours (i.e. toxic). This is especially important given that early research in the coach behaviour domain identified that punitive behaviours can have negative impacts on athlete self-esteem and anxiety.^1,59^

Coaches discussed that they employed toxic behaviours to *emphasize winning, promote competitiveness, and light a fire*. Toxic behaviours and abusive supervision were originally conceptiualized in organizational research.^60^ In sport research, few studies have examined these types of coaching behaviours. Bartholomew and colleagues^61^ found examples of behaviours from British coaches and youth athletes across numerous sports (i.e. swimming, squash, and dancing) that parallel the toxic behaviours displayed by some coaches in the present study. Specifically, Bartholomew et al.^61^ discussed two behaviours (i.e. promoting ego involvement and intimidation behaviours) used by coaches to demonstrate their superiority towards athletes. Similarly, coaches in this study discussed two objectives (i.e. emphasizing winning and breeding competitiveness) that relate very closely to promoting ego involvement. Secondly, intimidation behaviours, were described by Bartholomew et al.^61^ as instances in which coaches use power or assertive techniques to promote the desired athlete behaviour such as *lighting a fire* in the present study, where coaches yelled or took an assertive tone with athletes. Bartholomew and colleagues contend that these types of behaviours can have negative implications on youth sport experiences by manifesting coach resentment, stunting autonomy, reducing motivation, and ultimately leading to burnout although the coaches in this study did not acknowledge potential negative outcomes of these behaviours, perhaps out of embarrassment or lack of awareness.

### Practical implications

The theoretical and methodological foundations of this study generate a number of practical implications. In particular, practical implications can be derived from our findings grounded on the following premise: (a) TFL has been shown to have direct applications for PYD, and (b) TFL interventions are known to be effective across a range of domains.^40^ Indeed, several researchers have identified that TFL behaviours have the ability to facilitate growth among followers. Interestingly, coaches in this study attempted to use TFL behaviours without any prior formal education. More importantly, the coaches explained that they commonly use TFL behaviours in an attempt to foster PYD outcomes. Coach developers and sport organizations might capitalize on this information by training youth sport coaches to more effectively use and recognize TFL behaviours.

To that end, researchers have successfully implemented transformational coaching workshops focussed on optimizing transformational coach behaviours which might be of value to coach developers.^27,31^ Sport organizations might also consider adopting a transformational approach at the organizational level in an effort to support coaches in fulfilling this mandate. For instance, by understanding coaches intentions, sport organizations can utilize behavioural economic principles—an approach to understanding decision making and behaviour—to promote or deter coaches from a certain set of behaviours that contribute to PYD.^11^ Specifically, programmes that adopt behavioural economic principles acknowledge that behaviour is influenced by contextual factors (e.g. psychological, cognitive, emotional, cultural, sociological), and that adopting a set of tools, such as development of incentives or establishing and communicating appropriate norms, can better fostor long-term behaviour change.^11^ In addition to targeting PYD outcomes with TFL behaviours, coaches with a better understanding of TFL may in turn be less likely to resort to transactional and toxic behaviours. Reducing negative behaviours could decrease young athletes’ exposure to a leadership style that can potentially limit the impact that sport participation can have on PYD.

On a methodological level, by combining systematic observation (i.e. the CLAS) and video-stimulated recall, the findings generated from this study have real-world implications. According to Turnnidge and Côté,^40^ by assessing coaches’ moment-to-moment application of leadership, the CLAS “offers practitioners a unique behavioural account of coaches leadership behaviours” (p. 223). In combination with a video-stimulated recall procedure, this study garnered accuracy and insight into participants’ thought processes, such as behavioural intentions, as they occured in a real-world setting.^46,62^ In sum, the collective advantages offered by the application of TFL, systematic observation, and video-stimulated recall in this study can serve to facilitate knowledge mobilization by strengthening our understanding of coaches’ intentions for the implementation of leadership behaviours in a real-world setting.

### Limitations and future directions

The current study sheds light on the applicability of transformational behaviours in youth sport; however, there were study limitations that can be addressed in future studies. First, this study examined only coach perceptions of leadership behaviours. In response, future studies may seek to obtain perceptions of athletes, parents, and/or other coaches to provide a richer understanding of leadership behaviours and their potential influence on youth development via similar methods. Secondly, although this study contained a sample of coaches who have a wide range of experiences in coaching, each coach was currently coaching a competitive level sports team between the ages of 13–18. Future research studies may seek to study the perception of coaches that work with athletes of different sports, ages, and competitive levels. Additionally, although female coaches were recruited for this study, none participated for the entirety of the study. It should be noted, however, that this sample of coaches could be considered relatively representative of the coach demographics for these sports, within this geographical region. Specifically, 89.5% of coaches in these organizations were male. Given that coaching and leadership literature report gender differences in leadership behaviours,^63^ future studies examining female coaches would be beneficial in the realm of coaching leadership research. Lastly, it is possible that there were differences in characteristics between coaches who agreed to participate (e.g. athlete-centred), versus coaches who did agree to participate in the study (e.g. win-at-all cost attitude), which would have resulted in a sampling bias. With this in mind, future research could examine the link between the coaching characteristics of youth sport coaches with various leadership stypes (e.g. transformational vs. toxic leadership).

## Conclusions

This study provides novel insight into youth sport coaches behavioural intentions during the implementation of leadership behaviours. Given that understanding the motivation behind coaching behaviours can improve the likelihood of implementation, this study can help guide researchers’ application and understanding of coach leadership behaviours in this context. Practically, sport programmes that focus on coach-athlete relationships by promoting transformational coaching behaviours contribute to the fulfilment of athletes’ personal assets, and by extension, enhance the impact that sport can have on PYD. Nonetheless, this is a need for more research in this area to further solidify our understanding of coach leadership in youth sport, therefore it is hoped that the findings from this study will spark further interest in this important area of research.
